# Pregnenolone sulfate induces transcriptional and immunoregulatory effects on T cells

**DOI:** 10.1038/s41598-024-57327-0

**Published:** 2024-03-21

**Authors:** Yasmine El Hajj, Tala Shahin, Mame Massar Dieng, Manar Alshaikh, Mostafa Khair, Vinu Manikandan, Youssef Idaghdour

**Affiliations:** 1 Program in Biology, Division of Science and Mathematics, New York University Abu Dhabi, Abu Dhabi, United Arab Emirates; 2 Core Technology Platforms, New York University Abu Dhabi, Abu Dhabi, United Arab Emirates

**Keywords:** Gene expression analysis, Gene regulation in immune cells

## Abstract

Pregnenolone sulfate is a steroid metabolite of the steroidogenesis precursor, pregnenolone, with similar functional properties, including immunosuppression. We recently reported an elevation in serum levels of pregnenolone sulfate in children with malaria, contributing to an immunosuppressed state. Yet, the molecular mechanisms in which this steroid exerts its immunoregulatory functions are lacking. In this study, we examined the effects of pregnenolone sulfate on T cell viability, proliferation and transcriptome. We observed a pregnenolone sulfate dose-dependent induction of T cell death and reduction in proliferation. RNA sequencing analysis of pregnenolone sulfate-treated T cells for 2 and 24 h revealed the downregulation of pro-inflammatory genes and the upregulation of the steroid nuclear receptor superfamily, NR4A, as early-response genes. We also report a strong activation of the integrated stress response mediated by the upregulation of *EIF2AK3*. These results contribute to the knowledge on transcriptional regulation driving the immunoregulatory effects of pregnenolone sulfate on T cells.

## Introduction

Steroids are hormones involved in regulating multiple biological functions ranging from behavior, fertility and metabolism, to inflammation and immune regulation^1^. They are used clinically as immunosuppressants to treat conditions including rheumatoid arthritis^2^, allergic asthma^3^ and organ transplantation^4^. Endogenous steroid synthesis, or steroidogenesis, commences with the conversion of cholesterol to the precursor steroid, pregnenolone, by the enzyme CYP11A1. This process takes place in the adrenal glands, gonads and placenta, producing downstream steroid hormones including corticosteroids, progesterone, estrogen and testosterone^5^. Local steroidogenesis of pregnenolone has been reported in lymphocytes^6–9^, adipocytes^10^, tumors^11^, and the nervous system^12^. As a neurosteroid, pregnenolone enhances cognition, learning and memory, promotes recovery after spinal cord injury, regulates myelin synthesis, restores motor function, improves depressive symptoms, and reduces stress and anxiety^13^. However, its production by immune cells, such as T helper 2 (Th2) cells, is associated with immunomodulatory functions^8^.

The immunosuppressive effects of pregnenolone actively restore homeostasis, limiting tissue damage and chronic inflammation after pathogen clearance^14^. Pregnenolone dampens macrophage secretion of pro-inflammatory cytokines via inhibition of signal activation by promoting the degradation of toll-like receptors, TLR2/4^15^, as well as blocking the binding of TLR4 to myeloid differentiation factor 2^16^. Moreover, it inhibits T cell proliferation and B cell immunoglobulin class switching through undefined mechanisms^1,8,17^. These effects can subvert immune cell function in the context of both cancer and infection. Tumor infiltrating T cells produce de novo pregnenolone, which results in the induction of the immunoregulatory M2 phenotype in macrophages, and suppression of natural killer and T cell function^1,9^. These properties, in addition to pregnenolone’s ability to bind to mutated androgen receptors, promote tumor growth in prostate cancer and melanoma^18^.

Pregnenolone sulfate is a conjugated metabolite of pregnenolone found circulating at higher concentrations in the serum due to its increased solubility and expedited transport relative to pregnenolone^19^. While some biochemical effects are different between both steroids, such as their binding to neurotransmitters, some functional properties are shared, including enhanced cognition and immunosuppression^8,13,17,20,21^. In a recent study on human malaria, we recorded elevated in vivo serum levels of pregnenolone sulfate, in children during *Plasmodium falciparum* infection, impacting gene expression of key regulators of lymphocyte activation and proliferation, and the attenuation of the host immune response^17^.

While there is evidence supporting the downstream consequences of pregnenolone sulfate in the context of inflammation, the molecular mechanisms mediating its effects on immune cell function are poorly understood. In this study, we investigated the effects of pregnenolone sulfate on T cell growth and proliferation and on the transcriptome of primary expanded T cells. We first established and validated the immunosuppressive properties of pregnenolone sulfate by conducting in vitro dose–response analysis, measuring T cell viability and proliferation. Then, we used RNA sequencing to analyze the genome-wide transcriptional response of expanded, TCR (T cell receptor)-activated or non-activated T cells, non-treated and treated with pregnenolone sulfate at two timepoints (2 and 24 h post-treatment). The analysis identified early response genes impacted by pregnenolone sulfate treatment and revealed the activation/inhibition status of pathways mediating the T-cell response and the observed immunosuppressive state.

## Results

**Figure 1:**
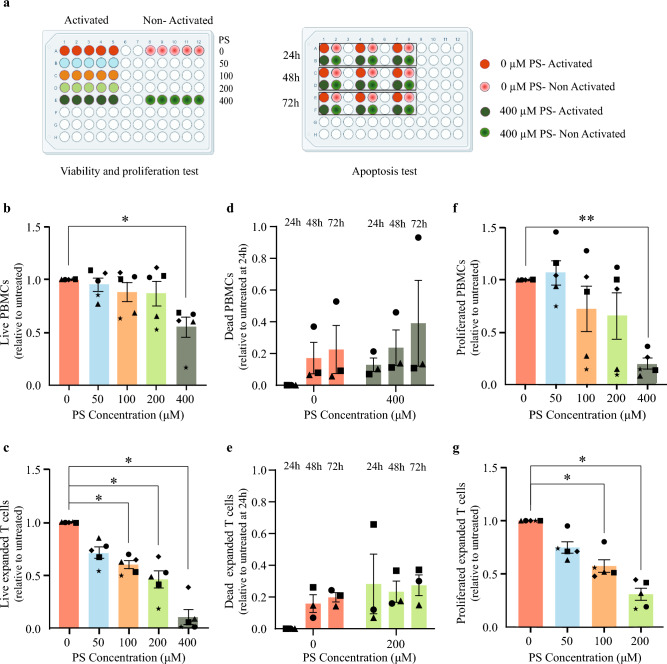
Effect of pregnenolone sulfate on cell viability and T cell proliferation in PBMCs and activated expanded T cells. (**a**) Experimental design of PBMC and expanded T-cell viability, proliferation and apoptosis assays. PBMCs and expanded T cells from 5 healthy donors were activated or non-activated with anti-CD3/anti-CD28 beads, and non-treated or treated with 50, 100, 200, or 400 μM of pregnenolone sulfate (PS) 24 h post-activation. Viability and proliferation were measured on day 6 for PBMCs and day 4 for expanded T cells post-activation. The apoptosis test was performed 24, 48, and 72 h after treatment using the same experimental design. All assays were measured using flow cytometry. Normalized bar plots showing for each of the PBMCs and expanded T cells respectively the (**b,c**) viability (7AAD^-^), (**d,e**) apoptosis (AnnexinV^+^PI^-^, AnnexinV^+^PI^+^, AnnexinV^-^PI^+^) and (**f,g**) proliferation (CFSE dye dilution) of TCR-activated cells upon treatment with the assigned concentration of pregnenolone sulfate. All recorded values plotted per donor are relative to that of the corresponding non-treated condition (and to the 24-h non-treated condition for the apoptosis test). Statistical analyses were performed using one-way ANOVA for the proliferation and viability assays, and two-way ANOVA for the apoptosis assay (*p < 0.05, **p < 0.01; otherwise, nonsignificant). Figure 1a was created using biorender.com.

Previous studies have demonstrated the inhibitory effect of pregnenolone and pregnenolone sulfate on T-cell proliferation^8,17^. We expand upon these studies and assess the effect of increasing concentration of pregnenolone sulfate on the viability, apoptosis and proliferation of both peripheral blood mononuclear cells (PBMCs) and primary expanded T cells following 24 h of TCR-activation with anti-CD3/anti-CD28 antibodies (Fig. 1a).

### Pregnenolone sulfate induces cell death in a dose-dependent manner

Viability and apoptosis assays were conducted to investigate the cytotoxicity of pregnenolone sulfate. We recorded a significant effect on cell viability due to treatment with 4 increasing concentrations of pregnenolone sulfate in the TCR-activated PBMC (p = 0.005; Fig. 1b, Supplementary Fig. S1) and expanded T cell samples (p = 0.03; Fig. 1c; one-way ANOVA, Supplementary Fig. S2) at days 6 and 4 post-TCR-activation, respectively. A marked decrease in viability was observed when cells were treated with the highest pregnenolone sulfate concentration (400 μM for PBMCs: p = 0.04; of 200 μM for expanded T cells: p = 0.01). This effect was also noted in the non-activated PBMC (p = 0.02; Supplementary Fig. S3a) and expanded T cell counterparts (p = 0.02; Supplementary Fig. S3b). Apoptosis assays were performed in activated and non-activated PBMCs and expanded T cells treated with 400 μM and 200 μM of pregnenolone sulfate, respectively, at the following time points post-pregnenolone sulfate treatment: 24, 48, and 72 h (two-way ANOVA test, Supplementary Fig. S4). In PBMCs, cell death increased by an average factor of 1.5 in activated, and 2.1 in non-activated pregnenolone sulfate-treated samples relative to the non-treated samples per time point, with the majority of the resulting dead cells being in the late apoptotic stage (Fig. 1d, Supplementary Figs. S3c, S4a and S5a–f). Similar trends were observed in the expanded T cell samples with average factors of 1.9 and 2.0 in activated and non-activated states, respectively (Fig. 1e, Supplementary Figs. S3d, S4b and S5g-l). Overall, we highlight an induction of cell death with decreasing viability in both cellular models consistent with increasing concentrations of pregnenolone sulfate treatment.

### Pregnenolone sulfate inhibits PBMCs and expanded T-cell proliferation

Next, we tested the effect of pregnenolone sulfate on the proliferation of live (7AAD^−^) T cells in the PBMC samples and observed a significant effect of the metabolite (p = 0.02, one-way ANOVA, Fig. 1f, Supplementary Fig. S1). The most pronounced effect was observed at the 400 μM concentration (p = 0.005, Fig. 1f). On average, 80% more live cells in the activated non-treated samples proliferated relative to the activated 400 μM pregnenolone sulfate-treated samples. In our expanded T-cell model, a significant impairment of T-cell proliferation was recorded upon pregnenolone sulfate treatment (p = 0.003), particularly at the 100 μM (p = 0.02) and 200 μM concentrations (p = 0.01, Fig. 1g, Supplementary Fig. S2). Proliferation at 400 μM of pregnenolone sulfate could not be measured as the number of live expanded T cells was insufficient due to high levels of cell death. Hence, our results indicate an inhibition in T-cell proliferation upon increasing concentrations of pregnenolone sulfate in both cellular systems, with expanded T cells showing heightened sensitivity to the treatment.

### Pregnenolone sulfate induces transcriptional changes promoting immunosuppression

**Figure 2:**
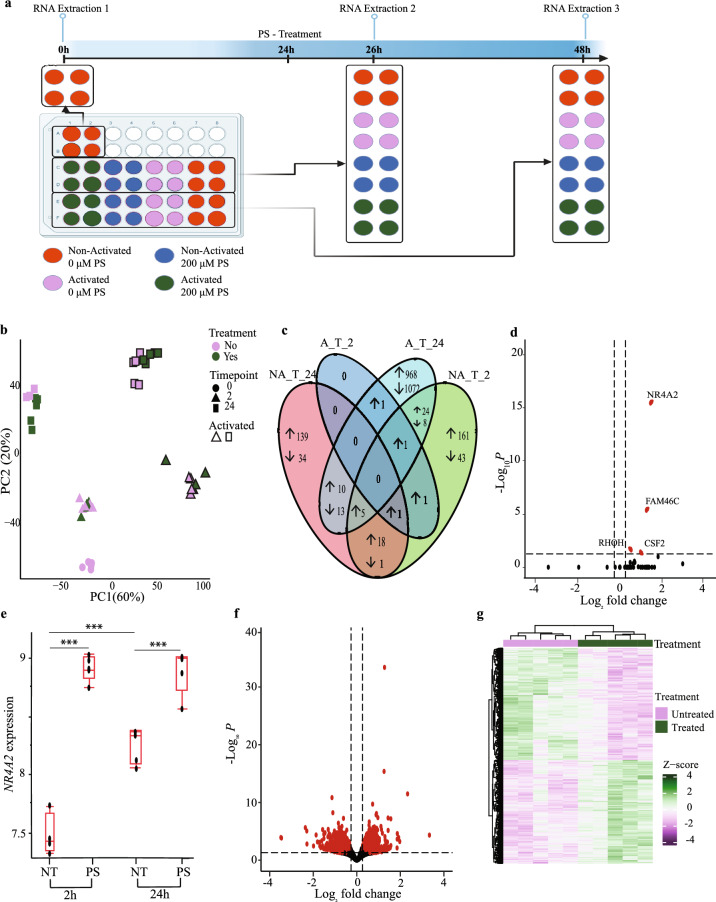
The transcriptomic signature of T cells treated with pregnenolone sulfate. (**a**) Experimental design. RNA was extracted from TCR-activated (24 h) and non-activated, pregnenolone sulfate (PS)-treated and non-treated samples at 2 h and 24 h post-treatment. (**b**) PCA of the transcriptome of 44 samples. (**c**) Venn diagram showing the number of differentially expressed genes (DEGs) across the 4 conditions (|fold change (FC)| ≥ 1.2, Benjamini–Hochberg (B-H) false discovery rate (FDR) < 0.05). (**d**) Volcano plot showing DEGs as a result of pregnenolone sulfate-treatment at 2 h post-treatment in TCR-activated samples. Significant DEGs after treatment (|FC| ≥ 1.2, B-H FDR < 0.05) are shown in red. (**e**) Box Plots showing the VST (variance stabilizing transformation)-normalized expression levels of *NR4A2* across conditions. Box plots show the median, the 25th and 75th percentiles as box edges, and the 5th and 95th percentiles as bounds of the whiskers. Two-way ANOVA test was performed and significance between treated or non-treated samples across and within time points is shown (***P *adj* < 0.0001). (**f**) Volcano plots and (**g**) heatmap showing DEGs upon 24 h of pregnenolone sulfate-treatment in TCR-activated samples (same statistical thresholds as in (d ). Heatmap generated in RStudio using the ComplexHeatmap package (v. 2.16.0)^22^.

In order to investigate the transcriptomic effects of pregnenolone sulfate that induce downstream immunosuppression, we performed bulk RNA sequencing of treated and non-treated expanded T cells. A pregnenolone sulfate concentration of 200 μM was chosen, based on the results of the dose–response analysis, as it led to downstream functional changes without detrimental effects on cell viability or changes in the CD4^+^ and CD8^+^ T-cell ratios compared to the untreated control (Fig. 2a, Supplementary Fig. S6). To query the effect of pregnenolone sulfate treatment in activated T cells (to simulate infection), RNA sequencing of treated and non-treated expanded T cells under TCR-stimulated and unstimulated states and at an early (2 h) and later (24 h) time points was performed (Fig. 2a).

Principal component analysis (PCA) of the full dataset revealed the relative contributions of TCR-activation, treatment and time on the correlation structure of the transcriptome and shows that PC1 and PC2 capture 80% of the variance. The clustering shows that activation has the strongest effect clearly captured by PC1 (60% of the variance) followed by the time point effect captured by PC2 (20% of the variance) (Fig. 2b). The effect of treatment on clustering is not as strong as that of activation and time, nonetheless it is readily visible at the 24-h time point in the PC plot, suggesting that differential expression is more pronounced at the 24-h time point relative to 2 h post-treatment.

As our interest lies in the effects of pregnenolone sulfate treatment on T cells upon activation, we focused on this effect and performed gene-by-gene differential expression analysis. This analysis revealed that four and 2102 genes are significantly differentially expressed (False Discovery Rate (FDR) < 5%, |fold change| ≥ 1.2) at the 2 and 24-h time points, respectively, which is consistent with the PCA and demonstrates the more pronounced effect of pregnenolone sulfate 24 h post-treatment (Fig. 2c and Supplementary Tables S1 and S2). To assess the magnitude of the effect of pregnenolone sulfate on the T cell transcriptome independently of activation, we performed the same analysis for the non-activated treated cells at the two time points. In contrast to the activated samples, this analysis revealed that 263 and 221 genes are significantly differentially expressed at the 2 and 24-h time points, respectively (Fig. 2c, Supplementary Fig. S7 and Tables S1 and S2). These results clearly show that the overall pregnenolone sulfate early and late (i.e. 2 and 24 h post-treatment respectively) inhibitory effects on the T cell transcriptome are activation-state specific.

Of the four upregulated genes at the 2-h time point post-pregnenolone sulfate treatment in activated T cells, *NR4A2*, an early response gene, was also found to be upregulated at 24-h together with *NR4A1* (Fig. 2d and e and Supplementary Tables S1 and S2). Moreover, a comparative analysis of the common early (2 h) pregnenolone sulfate response genes between activated and non-activated T cells reveals *NR4A2* to be upregulated in both groups, with *NR4A1* and *NR4A3* additionally upregulated at 2 h of treatment in non-activated T cells (Supplementary Table S3). These genes are members of the NR4A family and part of the thyroid/steroid nuclear receptor superfamily, with roles in maintenance of T-cell homeostasis^23^. Moreover, *CSF2*, *RHOH* and *FAM46C,* involved in anti-proliferation and pro-apoptotic effects^24^, were also upregulated at the 2-h time point in activated T cells. Hence, the upregulation of the early response *NR4A2* gene 2 h after pregnenolone sulfate treatment regardless of TCR-activation, validated by real-time quantitative PCR (RT-qPCR; Supplementary Fig. S8), suggests its involvement in the onset of an anti-inflammatory transcriptional signature that would dictate downstream changes in T-cell function.

**Figure 3:**
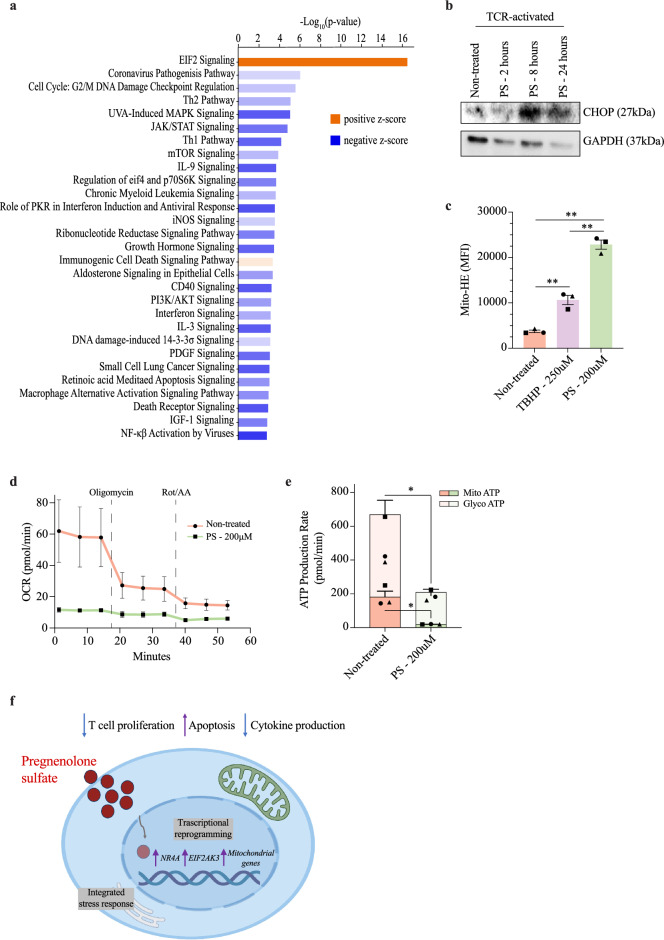
Ingenuity pathway analysis of pregnenolone sulfate-induced differentially regulated genes. (**a**) IPA canonical signaling pathway enrichment analysis of differential expression of the pregnenolone sulfate treatment effect at the 24 h time point. The strength of inhibition or activation of each signaling pathway is shown in colors corresponding to IPA activation/inhibition *z*-scores. Dark orange and dark blue indicate strong activation and inhibition predictions, respectively. (**b**) Western blot analysis of whole-cell lysates from expanded T cells of one donor that were TCR-activated and either non-treated, or treated with 200 μM of pregnenolone sulfate (PS) for 2, 8, or 24 h, run on a gel and blotted with CHOP and GAPDH antibodies. The original blots are presented in Supplementary Figure S10 (**c**) Bar graph showing the mean fluorescence intensity (MFI) of the Mito-HE dye staining expanded T cells from three donors (represented by the different shapes) that were activated with CD3/CD28 beads for 24 h and either non-treated, treated with 250 μM of TBHP for 1 h or with 200 μM of PS for 2 h. (**d**) Extracellular flux analysis with the graph showing oxidative consumption rate of expanded T cells from three donors that were activated with CD3/CD28 beads for 24 h, followed by no treatment or treatment with 200 μM of PS for 24 h and subjected to the ATP rate test. (**e**) Same ATP rate test as in (**d**) showing the mitochondrial (dark colors) and glycolytic (light color) ATP production rate from non-treated and treated T cells (individual donors represented by different shape). Paired *t*-test was performed on (**c**) and (**e**); *p < 0.05, **p < 0.01, ***p < 0.001. (**f**) Summary illustration of the effect of pregnenolone sulfate on the T cell transcriptome and effector functions.

To better capture the effect of pregnenolone sulfate on the co-regulation of gene networks at the 24-h time point in the TCR-activated group (Fig. 2f and g), we performed pathway enrichment using Ingenuity Pathway Analysis (IPA). Several immune pathways were shown to be suppressed, including Th1 and Th2 pathways, cytokine (IFN, IL2, IL3, IL6, IL9, IL12 and IL23) as well as cytokine/antigen receptor signaling pathways (JAK/STAT, PI3K/AKT, mTOR and NFκB; Fig. 3a and Supplementary Table S4). Some of the genes involved in these pathways and downregulated as a result of pregnenolone sulfate treatment include *JAK1-3*, *STAT1-3*, *STAT6*, *IL10RA*, *IL6R*, *IL12RB1*, *IL18R1*, *PIK3CB*, *PIK3CD*, *PIK3CG*, *TBX21*, *IRF1*, *OAS1*, and *IKBKE* (Supplementary Tables S2 and S4). To verify the changes in expression of a selected number of genes, we performed RT-qPCR on *IL18R1*, *STAT3*, *NFATC2, SOCS1, IL2RB, CD40LG* and *TBX21* (Supplementary Fig. S9 and Table S5). RT-qPCR analysis has confirmed the trends observed in the RNA-sequencing analysis for the seven selected genes, including reduced gene expression in pregnenolone sulfate-treated activated expanded T cells at the 24-h time point. Moreover, upregulation of the expression of anti-inflammatory genes, *TSC22D3*, encoding the glucocorticoid-induced leucine zipper (GILZ), as well as mRNA degraders *ZFP36* and *ZFP36L2* was recorded (Supplementary Table S2), highlighting the overall induction and regulation of an immunosuppressed state^25^. A comparative analysis of the pregnenolone sulfate late (24-h) response genes between activated and non-activated T cells has revealed relatively fewer number of shared genes. These included *HOMER1*, *GADD45B*, *JUNB* and *NPRL2* involved in regulation of cell growth, as well as the aforementioned genes, *TSC22D3*, *ZFP36* and *ZFP36L2* (Supplementary Table S3).

To investigate whether pregnenolone sulfate mediates an immunosuppressed state through an impairment of downstream TCR signaling pathways, we tested the effect of T cell activation by comparing activated and non-activated T cells in the following four groups independently: 2-h and 24-h treated or non-treated groups. In the 2-h treated and non-treated groups, differential gene expression analysis comparing activated and non-activated T cells recorded 6,541 and 6,144 deregulated genes, respectively, whereas in the 24-h treated and non-treated groups, 5,194 and 4,074 deregulated genes were recorded, respectively (FDR < 5%, |fold change| ≥ 2; Supplementary Tables S6 and S7). The number of common deregulated genes between treated and non-treated groups at each time point is quite large, being 4,724 and 2,636 at 2 and 24 h respectively. Pathway enrichment analyses of the common deregulated genes using IPA at both time points has highlighted the activation of DNA replication and cell cycle pathways driven as a result of TCR activation (Supplementary Tables S8 and S9). However, enrichment analysis of the deregulated genes specific to the 2-h treated group revealed an activation of mitochondrial respiratory chain and inhibition of mitochondrial dysfunction pathways, not observed in the analysis of unique deregulated genes from the comparison of the 2-h non-treated group (Supplementary Table S8). Similarly, unique deregulated genes in the 24-h treated group specifically highlighted an inhibition of the TCR signaling pathway (z-score =  − 5.284; p-value = 0.013; Supplementary Table S9). Given that T cells were treated 24 h after TCR activation, these results support the dynamic regulatory effects pregnenolone sulfate has on activated T cells, starting with mitochondrial respiration, followed by downstream TCR signaling pathways. They also further emphasize that the regulatory effects of pregnenolone sulfate are activation-state dependent.

### Pregnenolone sulfate induces transcriptional changes promoting cellular stress

While the overall trend of the effect of pregnenolone sulfate treatment on T cells is inhibition of transcriptional pathways, the “EIF2 pathway” was significantly activated (z-score = 2.887, p = 3.56 × 10^–17^, Fig. 3a). A closer look into the differentially regulated genes within this pathway reveals the upregulation of the endoplasmic reticulum (ER) stress-induced eukaryotic translation initiation factor 2-alpha kinase 3 (*EIF2AK3*). The encoded protein, EIF2AK3, phosphorylates and inhibits EIF2A (upregulated) to trigger the integrated stress response^26,27^. This is in line with the upregulation of mRNA stability regulators and degraders (*ZFP36*, *ZFP36L2*, *YTHDF1*, *YTHDF3*, *RC3H1*, *CNOT8*, and *ELAVL1*), the cytoplasmic stress granule regulator, *ZFAND1* as well as the pathway’s downstream transcription factor target, *DDIT3*, encoding CHOP (Supplementary Table S2). Indeed, we observed an increase in the expression of the CHOP protein along the time-course of pregnenolone sulfate treatment of activated T cells (Fig. 3b, Supplementary Fig. S10). This indicated a specific activation of the EIF2AK3 integrated stress-response pathway, and not through IRE1, as we did not observe an increase in the downstream splicing of XBP1 (Supplementary Fig. S11).

Interestingly, and as observed in our differential expression analysis of activated to non-activated T cells treated with pregnenolone sulfate for 2 h, several mitochondrial oxidative phosphorylation subunits (*COX6A1*, *NDUFS3*, *NDUFV2*, *NDUFB10*, *NDUFC1*, and *SDHB*) and mitochondrial membrane transport genes (*VDAC1*, *FIS1*, *TIMM13*, *TIMM17A*, *TIMM44*, *TOMM20*, *TOMM40*) were upregulated as a result of the 24-h pregnenolone sulfate treatment in activated T cells (Supplementary Table S2). We speculated that this could be an effect of mitochondrial dysfunction. Therefore, we measured mitochondrial superoxide production and performed an extracellular flux assay to measure the ATP produced by both mitochondrial respiration and glycolysis. Analysis of the results has revealed significantly higher mitochondrial superoxide in pregnenolone sulfate-treated cells at 2 h (Fig. 3c, Supplementary Fig. S12). Moreover, a severe disruption in oxidative phosphorylation, as well as in glycolysis, of TCR-activated T cells treated with pregnenolone sulfate for 24 h was recorded, measured by the significantly reduced oxygen consumption and amount of mitochondrial and glycolytic ATP produced compared to the non-treated counterpart (Fig. 3d and e). Hence, the upregulation of stress-related and mitochondrial genes is likely a result of the ER and oxidative stress that cells treated with pregnenolone sulfate are enduring.

Overall, we observed the suppression of inflammatory pathways and activation of stress pathways under the effect of pregnenolone sulfate which render T cells in a hyporesponsive state in the presence of activating stimuli (Fig. 3f). The activation of the integrated stress response and oxidative stress are likely the drivers of the apoptosis observed upon pregnenolone sulfate treatment (Fig. 1d,e and Fig. S1c,d).

## Discussion

As a precursor of steroidogenesis and as a molecule synthesized and secreted by subsets of lymphocytes, the immunoregulatory properties of pregnenolone and its metabolites warrant deeper characterization. In this study, we investigated the effects of pregnenolone sulfate, the more soluble and abundant metabolite of pregnenolone, on the T-cell transcriptome and downstream molecular processes impacted. We chose a treatment concentration range of 50-400 μM, which is higher than the physiological in vivo human serum concentration, as these concentrations have been previously tested to exert their in vitro effects on T cells, considering the possibility of a lower pregnenolone sulfate uptake and thereby a lower intracellular working concentration^8,17,19,28^. By performing dose-escalation experiments in primary and in vitro expanded T cells, we first showed apoptotic effects that are heightened at increasing concentrations of pregnenolone sulfate, coupled with proliferation inhibition. We have previously shown that treatment of primary T cells with 400uM of pregnenolone sulfate leads to a significant reduction in both cell proliferation and the production of cytokines including IL-5, IL-10, IL-13, IFNγ and TNF^17^. Furthermore, Mahata et al. focused on pregnenolone’s effect on murine Th1, Th2 and B cells and found that pregnenolone treatment inhibits the proliferation of Th1 and Th2 cells, and causes a dose-dependent reduction in class-switching of B cells to IgG1 and IgE-producing cells^8^. Our results are consistent with the findings of these studies, confirming the shared functional effects of pregnenolone sulfate and pregnenolone, as well as the pregnenolone sulfate dose-dependent inhibition of downstream T-cell functions that promote the immunosuppressive effects of the conjugated steroid metabolite.

Our transcriptomic analysis to dissect the molecular events that lead to an immunosuppressed state upon pregnenolone sulfate treatment highlighted the propensity of the transcriptional effect in the context of TCR-activation, portrayed by the large increase in the number of differentially expressed genes from 2 to 24 h post-treatment. Meanwhile, the larger number of differentially expressed genes at the 2-h time point in the non-activated state suggests that pregnenolone sulfate also has a modulatory role on the T cell state that is transcriptionally achieved in a shorter period of time due to the cell’s quiescence, upon which larger effects might be observed in the case of activation of those T cells. Moreover, our analysis revealed *NR4A1* and *NR4A2* as early response genes in the cascade to immunosuppression. NR4A transcription factors are involved in the regulation of several T-cell effector functions. *Nr4a1*-deficient mice show increased CD4^+^/CD8^+^ T cell proliferation and antigen-specific CD8^+^ T cell expansion, higher glycolysis and respiration rate, as well as enhanced cytokine production, whereas *Nr4a1* overexpression in CD4^+^ T cells induced the upregulation of anergy genes^29–31^. Moreover, *Nr4a2*^-/-^ CD8^+^ CAR T cells showed reduced expression of exhaustion markers and demonstrated higher tumor regression and enhanced survival in tumor-bearing mice^32^. Hence, we speculate that the early expression of *NR4A* genes, together with *TSC22D3* encoding GILZ, sets the transcriptional reprogramming of T cells, enabling the suppression of downstream proliferation and effector functions. This is strengthened by our findings that show an inhibition of key growth and cytokine signaling pathways, differential expression of epigenetics remodelers, as well as higher expression of exhaustion markers (*RGS1*) and the anti-inflammatory cytokine mRNA degrader genes *ZFP36* and *ZFP36L2* upon pregnenolone sulfate-treatment^33,34^. However, knocking out *NR4A1* and/or *NR4A2* in these expanded T cells will likely give greater insights into their roles as early response regulators in the context of pregnenolone sulfate treatment.

From our analysis of gene networks that were activated as a result of pregnenolone sulfate treatment, we highlighted the involvement of the integrated stress response, mediated by the upregulation of *EIF2AK3.* EIF2AK3 phosphorylates and inhibits EIF2A resulting in a global translational reduction, together with upregulation of downstream *DDIT3* and the encoded protein, CHOP, selective translation of specific mRNAs leading to ER stress, cell cycle inhibition and eventually apoptosis^26^. Moreover, we observed an upregulation in several nuclear mitochondrial genes that suggest an attempt of T cells to go through metabolic rewiring to regain homeostasis as a result of pregnenolone sulfate treatment. This was highlighted in our results by an increase in mitochondrial superoxide production and respiratory dysfunction of TCR-activated T cells. These findings are of interest as they point to a previous study that investigated pregnenolone-interacting proteins in CD8^+^ T cells, where 70% of these proteins are localized in mitochondrial or ER membranes^28^. Thus, future studies that investigate whether pregnenolone sulfate’s non-genomic interactions drive the transcriptional changes observed, or vice-versa, would be revealing to better understand the mechanism of action of this metabolite. Moreover, transcriptional and epigenetic profiling of treated activated T cells at more time points to assess the dynamic genomic changes between the 2 and 24-h time points would further add to our understanding of the gradual changes and early responses to pregnenolone sulfate treatment.

All together, we have characterized the transcriptional response induced by pregnenolone sulfate in activated T cells, highlighting the pathways involved in its promotion of an immunosuppressive state together with its induction of metabolic reprogramming. In some cases, pregnenolone and its metabolites’ neuroprotective properties have been beneficial for enhancing learning and memory disorders, as well as modulating cognitive brain function. Given that pregnenolone sulfate is one of the body’s natural prevalent steroids, further studies to understand the precise mechanisms by which it exerts its immunomodulatory properties would enable its multipurposing to treat inflammatory disorders.

## Methods

### Donor information

In this study, venous blood was given by five donors to isolate PBMCs, whereby three were males and two were females, all between the age of 21 and 50 years and of Middle-Eastern, Asian or African descent. The five donors were healthy, hence they did not have any known significant health problems. Informed consent was obtained from the donors and all experimental protocols were approved by the Institutional Review Board of the New York University of Abu Dhabi and are in agreement with relevant guidelines and regulations.

### Isolation of PBMCs

Venous blood samples (15 mL) were collected from the 5 healthy donors in heparin tubes. PBMCs were isolated using the density gradient cell separation medium Histopaque™1077 (Sigma-Aldrich) according to the manufacturer's protocol. PBMCs were cultured in complete RPMI-1640 medium (Gibco) supplemented with 10% fetal bovine serum and 1% of penicillin–streptomycin (ThermoFisher Scientific) in a 5% CO_2_ incubator at 37 °C.

### T cell expansion

Freshly collected PBMCs from the five donors were plated in 48-well plates (1 million cells/well) containing T cell media: RPMI-1640 medium (Gibco) with 5% human serum (Sigma-Aldrich), 1% Pen-Strep (Thermo Fisher Scientific), 1X MEM non-essential amino acids solution (Sigma-Aldrich), 1X sodium pyruvate (Gibco), and 1X HEPES buffer (Sigma-Aldrich), in addition to 1.6μL of Phytohemagglutinin-L (PHA-L) per well (500X, Invitrogen; Cat. no.: 00-4977-93) and 20 ng/mL of IL2 (BioLegend), and placed in a 5% CO_2_ incubator at 37 °C. Cells were split every 2–3 days depending on density, and IL2 was replenished at a concentration of 20 ng/mL on the first two splits, then downscaled to 10 ng/mL. Cells were expanded for 12 days and were validated by flow cytometry using CD3-PE, CD4-PE and CD8-PB antibodies (Supplementary Fig. S6).

### Proliferation and viability assays

PBMCs and expanded T cells from the five healthy donors were counted (150,000 cells/well on a 96-well plate) and stained for proliferation assay with the CellTrace™ CFSE dye (ThermoFisher Scientific) in 1X Phosphate Buffered Saline (PBS) solution for 7 min, then blocked for 1 min with 1 mL of cold fetal bovine serum (FBS) on ice. Cells were then washed twice and resuspended in their respective culturing media, followed by plating 150μL of 150,000 cells into each well. PBMCs were TCR-activated using 1 μg/mL of anti-CD28 antibodies (eBioscience) in a 96-well polystyrene treated and non-pyrogenic round-bottom plate (Costar) coated with 1 μg/mL of anti-CD3 antibodies (clone OKT3, BioLegend). For expanded T cells, they were TCR-activated with 5μL CD3/CD28 Dynabeads™ (ThermoFisher Scientific) in T cell media with 10 ng/mL of IL2 and plated onto the aforementioned 96-well plates. Cells were subsequently treated with 50μL pregnenolone sulfate 24 h after TCR-stimulation (final concentrations of 50, 100, 200, and 400 μM of pregnenolone sulfate in RPMI/methanol media; Pregnenolone sulfate sodium salt (white solid powder) from TOCRIS, Cat. no. 5376, Batch No: 5A/262294, M.W.: 450.04 g/mol). Pregnenolone sulfate was weighed and solubilized in 100% methanol followed by complete media at a 1:4.6 ratio of methanol: complete media. Serial dilutions were performed to obtain decreasing concentrations of the steroid metabolite. Pregnenolone sulfate-free methanol/RPMI media was added to non-treated cells while preserving the same 1:4.6 ratio of methanol: complete media (final concentration of methanol in all conditions is ~ 5%, which is tolerated by cells). Cells from PBMCs and expanded T cells were incubated for 6 and 4 days respectively, after which the dilution of the CFSE dye was measured by flow cytometry to assess the proliferation of T cells.

Alongside the proliferation assay, we measured cellular viability, whereby half of the cultured cells were collected and stained with 3 μl of 7-amino-actinomycin D viability probe (7-AAD, BD Bioscience) and incubated for 20 min at 4 °C at the 6- and 4-day mark for PBMC and expanded T cell assays respectively. Flow cytometry buffer (300 μl, 1 × PBS with 2% FBS) was then added to the cells before flow cytometry analysis. For both proliferation and viability assays, cells were acquired on FACSAria™ III and FACSCanto™ instruments (BD Biosciences). Data was analyzed using FlowJo v10.8.1 software (BD Bioscience) and GraphPad Prism 9. The values (both proliferation and viability) recorded for each individual treated at the various concentrations of pregnenolone sulfate are relative to, and hence divided by, the value recorded from that individual in the untreated condition.

$$\mathrm{Relative\, proliferated}/\mathrm{live\, cells }=\frac{Value\, per\, individual}{Untreated\, value\, of\, same\, individual}$$

### Apoptosis assay

Apoptotic cells were measured 24, 48 and 72 h post-treatment with pregnenolone sulfate and detected using Annexin V-FITC Apoptosis Detection Kit (BD Pharmingen). Cells were stained with Annexin V-FITC (5 μL/test) and propidium iodide solution (PI, 5 μL/test), and then acquired on FACS Aria™ III (BD Biosciences). Data was analyzed using FlowJo v10.8.1 software (BD Bioscience) and GraphPad Prism 9. The total percentage of cells in the early apoptotic (AnnexinV^+^PI^-^), late apoptotic (AnnexinV^+^PI^+^) and necrotic (AnnexinV^-^PI^+^) lymphocyte gates were considered apoptotic (Supplementary Fig. S4). To calculate the relative value of dead cells, the total percentage of apoptotic cells recorded for each individual per time point treated at the various concentrations of pregnenolone sulfate are relative to, and hence divided by, the value recorded from that individual in the untreated condition at the 24-h time point, subtracted by 1.

$$\mathrm{Relative\, dead \,cells}= \left(\frac{\%\, total\, apoptotic\, cells\, per\, individual}{\%\, total\, apoptotic\, cells\, of \,untreated\, sample\, at\, 24\, hours\, from\, same\, individual}\right) -1$$

### Flow cytometry gating strategies

For the viability and proliferation experiments, PBMCs and expanded T cells were first gated according to forward scatter (FSC) and side scatter (SSC) (Supplementary Figs. S1, S2). From the obtained populations, live cells were gated using 7-AAD from which proliferated cells were quantified using FITC CFSE stains. For the apoptosis assay, PBMCs and expanded T cells were first gated according to FSC and SSC (Supplementary Fig. S4). From the obtained populations, PI and FITC Annexin V were used to quantify the number of live and apoptotic cells. AnnexinV^+^ PI^-^, AnnexinV^+^ PI^+^, AnnexinV^-^PI^+^ lymphocytes and expanded T cells were considered apoptotic. For the mitochondrial superoxide assay, expanded T cells were first gated according to FSC and SSC, followed by live cell gating on the SYTOX Red^-^ population (Supplementary Fig. S11). From the obtained populations, the mean fluorescence intensity was calculated for Mito-HE. All flow cytometry plots for gating strategies were produced using FlowJo v10.8.1 software (BD Bioscience).

### RNA-sequencing and analysis

Total RNA was isolated using the miRNeasy^®^ Mini kit (Qiagen, according to the manufacturer's protocol) and quantified using a Qubit instrument. In total, 44 cDNA libraries were generated by Juno™ system using the Advanta™ RNA-sequencing NGS Library Prep Kit and the 48.Atlas™ IFC (Fluidigm), quality-checked using a Bioanalyzer 2100 instrument, quantified using qPCR and followed by sequencing on a NextSeq instrument (Illumina). Quality control of paired-end raw sequencing reads generated from 44 samples was performed using FastQC (v0.11.5). Low-quality reads, sequencing adapters and overrepresented K-mers were removed using Trimmomatic (v0.32). The reads were aligned to the Human reference genome (Ensembl release 84-GRCh38) using the STAR aligner (v2.5.2a) and default alignment parameters to produce BAM files. HTSeq-Count (v0.6.1p1) was then used to generate read counts per gene based on the 84-GRCh38 GTF considering that the RNA-sequencing library is stranded. The counts generated were then filtered to remove unexpressed or very lowly expressed genes. The full bioinformatic analysis procedure was conducted as previously described^35^. Differential expression analysis was performed using DESeq2 where treated and non-treated samples were contrasted across the following conditions: (1) non-activated non-treated samples marking the basal time point (0 h, n = 6), (2) activated samples 2 h post-treatment with pregnenolone sulfate (n = 5 treated, n = 4 non-treated), (3) activated samples 24 h post-treatment with pregnenolone sulfate (n = 5 treated, n = 5 non-treated), (4) non-activated samples 2 h post-treatment with pregnenolone sulfate (n = 5 treated, n = 5 non-treated) and (5) non-activated samples 24 h post-treatment with pregnenolone sulfate (n = 6 treated, n = 3 non-treated). An adjusted p-value of 0.05 (5% FDR using the Benjamini–Hochberg procedure) and an absolute fold change (shrunken using apeglm implemented in DESeq2) threshold of 1.2 were used to determine significance of differential expression. Gene enrichment analysis was conducted using Ingenuity Pathway Analysis (IPA, Qiagen).

### RT-qPCR validation of RNA-sequencing data

RT-qPCR assays (ThermoFisher Scientific) were used to validate the expression levels of seven T cell function genes: *STAT3* (Hs00374280_m1), *TBX21* (Hs00894392_m1), *NFATC2* (Hs00905451_m1), *CD40LG* (Hs00163934_m1), *SOCS1* (Hs00705164_s1), *IL18R1* (Hs00977691_m1) and *IL2RB* (Hs01081697_m1), as well as 2 NR4A family genes: *NR4A1* (Hs00374226_m1) and *NR4A2* (Hs01117527_g1, all Thermo Fisher Scientific). Total RNA was used from up to 32 samples, including treated/non-treated with pregnenolone sulfate and TCR-activated/non-activated, at time points 0, 2, or 24 h. Reverse transcription was done using 160 ng of total RNA and 1 µl of reverse-transcription master mix in a total reaction volume of 5 µl followed by a standard Flex Six™ Gene Expression IFC dynamic array gene expression TaqMan assay workflow (PN 100–7070, Fluidigm) using a BioMark HD instrument (Fluidigm, as per the manufacturer’s protocol). Relative expression levels were measured using the 2^–ΔΔCt^ procedure and normalized using the housekeeping genes ACTB (4448892, Thermo Fisher Scientific) and GAPDH (4331182, Thermo Fisher Scientific).

### Western blot analysis

Expanded T cells were plated at a density of 1.5 × 10^6^ cells/well in 1.5 mL of T cell media (supplemented with 10 ng/mL of IL2) on a 24-well plate and activated with 25 μl/well of CD3/CD28 DynaBeads™ (ThermoFisher Scientific) for 24 h. Cells were then either non-treated or treated with 200 μM of pregnenolone sulfate for 2, 8 or 24 h. After each time point, cells were harvested in 15 mL falcon tubes, centrifuged at 300 g for 5 min, washed with ice cold PBS, and lysed in RIPA buffer (150 mM NaCl, 1% Nonidet P-40, 0.5% DOC, 0.1% SDS, 50 mM Tris (pH 7.4)) supplemented with protease inhibitor (Sigma Aldrich) and 1 mM Na_3_VO_4_ (Thermo Fisher Scientific). Lysates were then centrifuged at 13,000 *rpm* for 10 min, and upon adding 1X Laemmli Sample Buffer (Biorad), were analyzed by running on a 10% Mini-PROTEAN^®^ TGX™ Precast Protein Gels (Biorad). This was followed by Western blotting with CHOP (Cell Signaling; D46F1; 1:1000 dilution in EveryBlot buffer (Biorad)) and glyceraldehyde-3-phosphate dehydrogenase GAPDH primary antibodies (Santa Cruz; 6C5; 1:1000).

### XBP1 splicing assay by PCR

Total RNA was extracted from expanded T cells that were seeded, TCR-activated and treated/non-treated in the same conditions as for Western blot analysis above using the RNeasy Mini kit (Qiagen). cDNA was then synthesized using the cDNA Preparation with Reverse Transcription Master Mix (Fluidigm, PN 100-6297), followed by PCR amplification using the KAPA HiFi HotStart ReadyMix PCR Kit (Kapa Biosystems), both according to manufacturers’ protocols. Primers (5’ to 3’) used for amplification of XBP1 splicing variants and housekeeping gene include: sXBP1 forward CTGAGTCCGAATCAGGTGCAG; uXBP1 forward: CAGCACTCAGACTACGTGCA; tXBP1 forward: TGGCCGGGTCTGCTGAGTCCG; reverse primer used for all XBP1 variants: ATCCATGGGGAGATGTTCTGG; HPRT forward: CCCTGGCGTCGTGATTAGTG; HPRT reverse: TCGAGCAAGACGTTCAGTCC. PCR products were then run on a 2% agarose gel and imaged on a ChemiDoc™ MP Imaging System (Biorad).

### Mitochondrial superoxide assay

Expanded T cells from three donors were plated at a density of 200,000 cells in 150 μl of T cell media (supplemented with 10 ng/mL of IL2) per well on a 96-well U-bottom plate and activated with 5 μl/well of CD3/CD28 DynaBeads™ (ThermoFisher Scientific). After 24 h, cells were either treated with 200 μM of pregnenolone sulfate for 2 h, 250 μM of tert-Butyl hydroperoxide (TBHP; Life Technologies™) for 1 h or left untreated. Following the treatments, cells were stained with Mito-HE (TOCRIS, Cat. no. 7641) at a final concentration of 5 μM for 20 min at 37 °C. Cells were then washed with PBS and stained with 5 nM SYTOX™ Red Dead Cell Stain (ThermoFisher Scientific, Cat. no. S34859) for 15 min at 37 °C and immediately acquired on a FACSCanto™ instrument (BD Biosciences).

### Extracellular flux analysis: real-time ATP rate assay

Expanded T cells from three donors that were activated for 24 h with 25 μl/1 × 10^6^ cell of CD3/CD28 DynaBeads™ (ThermoFisher Scientific) were then non-treated or treated with 200 μM of pregnenolone sulfate for another 24 h. Expanded T cells were then counted and adjusted to 250,000 cells per 180 µl of warm assay media: Agilent Seahorse XF DMEM medium pH 7.4 (Agilent, Part no. 103575-100) supplemented with 1 mM XF pyruvate, 2 mM XF glutamine and 10 mM XF glucose (all from Agilent). On a Seahorse XFe/XF96 cell culture microplate, 180 μl of cells were loaded and centrifuged to form a monolayer at 200 g for 3 min. Cells were then placed in a non-CO_2_ incubator for 1 h at 37 °C. Meanwhile, oligomycin together with rotenone/antimycin A (provided in the XF Real-Time ATP Rate Assay kit, Agilent) were resuspended in assay media to get 150 μM and 50 μM stock concentrations, respectively. A 10X solution of each of oligomycin and rotenone/antimycin A was then prepared in assay media, whereby 20 μl and 22 μl of each dilution was loaded onto ports A and B of the hydrated XF sensor cartridge (Agilent) to make a final concentration of 1.5 μM and 0.5 μM of each compound, respectively, when dispensed into the cell wells. The cartridge was then loaded into the Seahorse XFe96 analyzer (Agilent) for calibration, followed by loading of the cell plate. Oxygen consumption rates (OCR) (pmol/min) and extracellular acidification rates (ECAR) (mpH/min) of cells in each well were measured using the ATP Rate assay template at 37 °C, whereby 3 cycle-measurements of 5 min each were done for basal respiration, after injection of oligomycin and after injection of rotenone/antimycin A. Mitochondrial ATP production rate is then quantified by the program as a result of the decrease in OCR following injection of oligomycin, an ATP synthase inhibitor. Glycolysis ATP production rate is also quantified through the measurement of ECAR following complete inhibition of mitochondrial respiration upon injection of rotenone/antimycin A.

### Statistical analysis

All statistical tests were performed in GraphPad Prism 9. For the proliferation and viability assays, the difference in percentages of TCR-activated live and proliferated cells non-treated and treated with different concentrations of pregnenolone sulfate were assessed using one-way ANOVA test (p < 0.05) followed by Tukey’s post-hoc comparison tests (p < 0.05). A paired *t*-test (p < 0.05) was used for the statistical analysis of treatment effect on cell viability in non-activated samples. For the apoptosis assay, the difference in the numbers of apoptotic cells between pregnenolone sulfate-treated and non-treated groups per time point was assessed using a two-way ANOVA test (p < 0.05). An unpaired t-test (p < 0.05) was used to indicate significance in the comparison of qPCR 1/ΔCt values for treated and non-treated samples and a two-way ANOVA for comparisons between treated or non-treated samples across and within time points. A paired *t*-test (p < 0.05) was used to assess the significance in the differences of values recorded for the Mito-HE assay and the ATP Rate assay.

## Limitations of the study

Although the general trend of changes in the expression levels of T cell genes indicates that pregnenolone sulfate affects multiple T cell types, further investigations are needed to uncover potential cell type- or state-specific effects of the steroid metabolite. In addition, while genes encoding enzymes that metabolize pregnenolone sulfate into downstream steroids were not found to be expressed in our expanded T cell samples, transcriptional effects due to steroid molecules synthesized from pregnenolone sulfate cannot be completely ruled out. Moreover, ascertaining differences in the functional and biochemical effects of pregnenolone sulfate in contrast to pregnenolone would require further investigation.

### Supplementary Information

Supplementary Figures.

Supplementary Tables.

## Data Availability

Processed RNA-sequencing data are openly available via Gene Expression Omnibus (Accession number, GSE225725). All original code is available at: 10.5281/zenodo.10842848, https://github.com/Yidaghdour/Pregnenolone-Sulfate-RNA-Seq/. Any additional information required to reanalyze the data reported in this paper is available from the lead contact upon request.
