# Supplementary material for: Pregnenolone sulfate induces transcriptional and immunoregulatory effects on T cells
Source: Sci Rep. 2024 Mar 21;14:6782. doi: 10.1038/s41598-024-57327-0 (PMC10958000; doi:10.1038/s41598-024-57327-0)
Supplement: Supplementary file 1 — Supplementary Figures. [file 41598_2024_57327_MOESM1_ESM.pdf]

Pregnenolone sulfate induces transcriptional and immunoregulatory effects on T cells

Yasmine El Hajj[1,+], Tala Shahin[1,+], Mame Massar Dieng[1], Manar Alshaikh[1], Mostafa Khair[2], Vinu Manikandan[1], Youssef Idaghdour[1,*]

Supplemental figures and legends

Figure S1

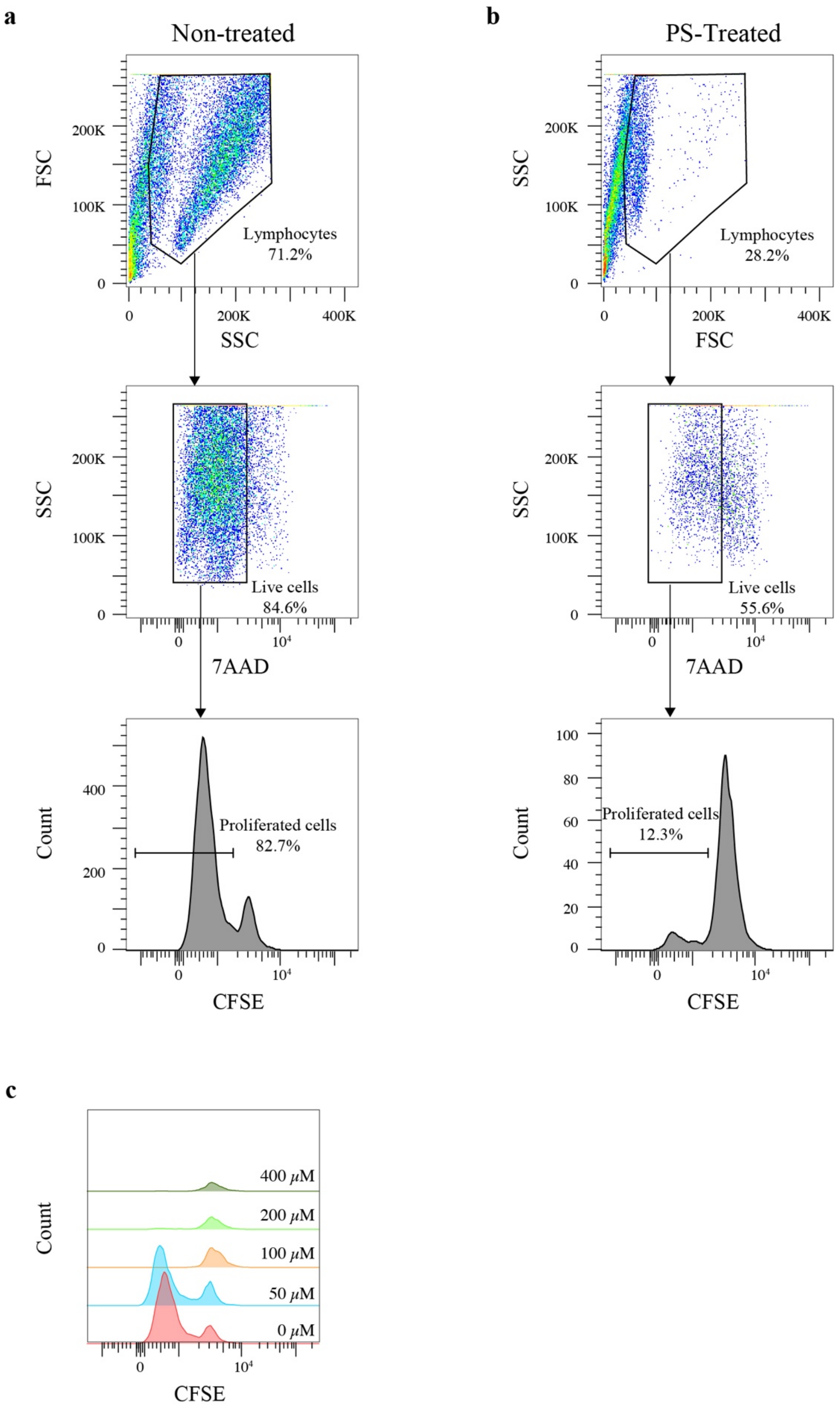

a
Non-treated
FSC
SSC
Lymphocytes
71.2%
7AAD
Live cells
84.6%
Count
CFSE
Proliferated cells
82.7%
b
PS-Treated
SSC
FSC
Lymphocytes
28.2%
7AAD
Live cells
55.6%
Count
CFSE
Proliferated cells
12.3%
c
400 μM
200 μM
100 μM
50 μM
0 μM
Count
CFSE

Figure S1. Gating strategy used in PBMC cell viability and proliferation assays. Flow cytometric plots showing the gating strategy used in the (**a**) non-treated and (**b**) 200 μM pregnenolone sulfate (PS)-treated PBMC cell viability and proliferation assays from one individual. Lymphocytes were gated according to FSC and SSC, and live cells were gated according to 7-AAD. CFSE-stained live cells were detected using CFSE FITC. (**c**) Flow cytometric plot of CFSE-stained live lymphocytes for the same individual under the indicated pregnenolone sulfate concentrations.

Figure S2

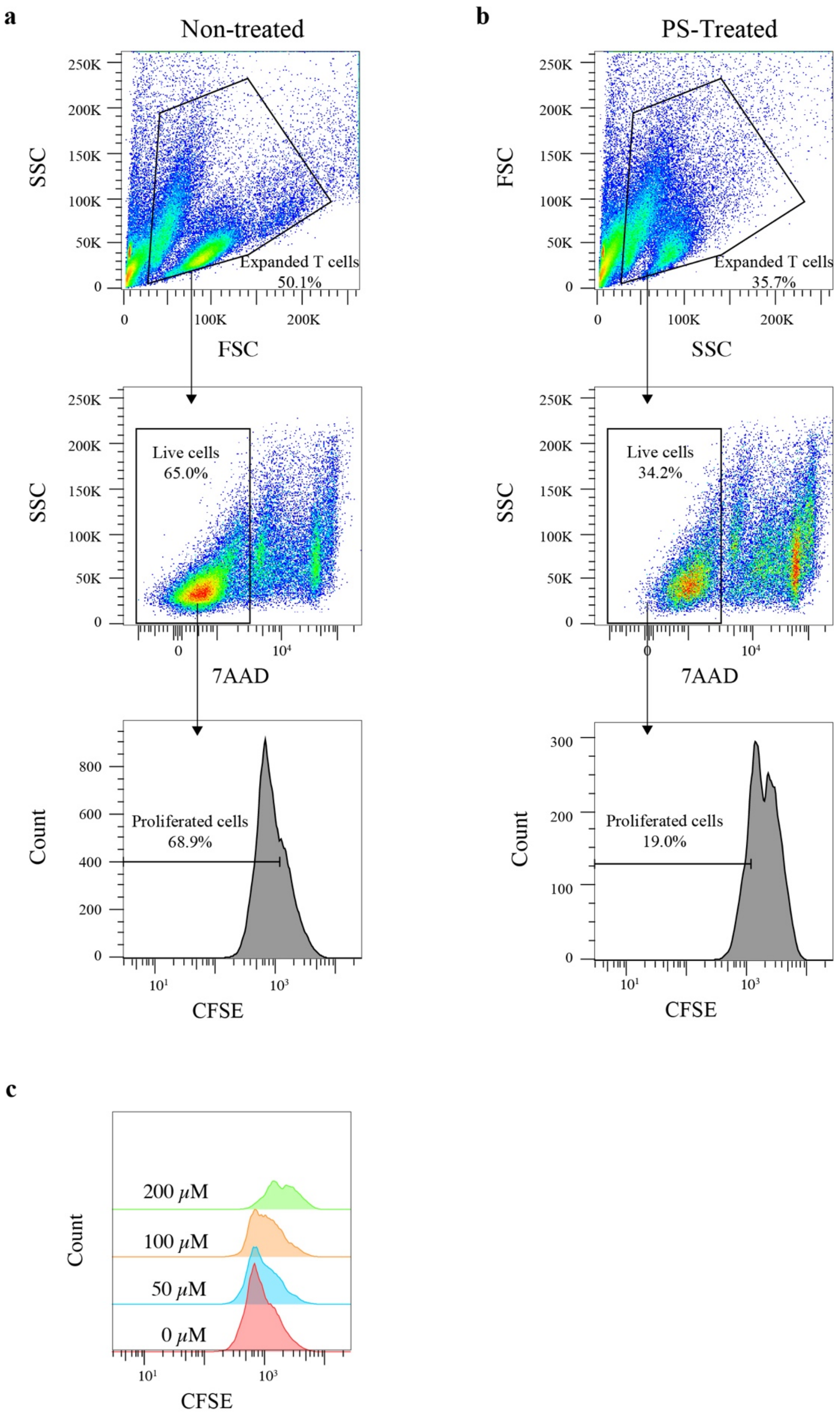

a
Non-treated
SSC
FSC
Expanded T cells
50.1%
Live cells
65.0%
7AAD
Count
Proliferated cells
68.9%
CFSE
b
PS-Treated
FSC
SSC
Expanded T cells
35.7%
Live cells
34.2%
7AAD
Count
Proliferated cells
19.0%
CFSE
c
200 μM
100 μM
50 μM
0 μM
Count
CFSE

Figure S2. Gating strategy used in expanded T cells viability and proliferation assays. Flow cytometric plots showing the gating strategy used in the (**a**) non-treated and (**b**) 200 μM pregnenolone sulfate (PS)-treated expanded T cells viability and proliferation assays from one individual. Expanded T cells were gated according to FSC and SSC, and live cells were gated according to 7-AAD. CFSE-stained live cells were detected using CFSE FITC. (**c**) Flow cytometric plot of CFSE stained live expanded T cells for the same individual under the indicated pregnenolone sulfate concentrations.

Figure S3

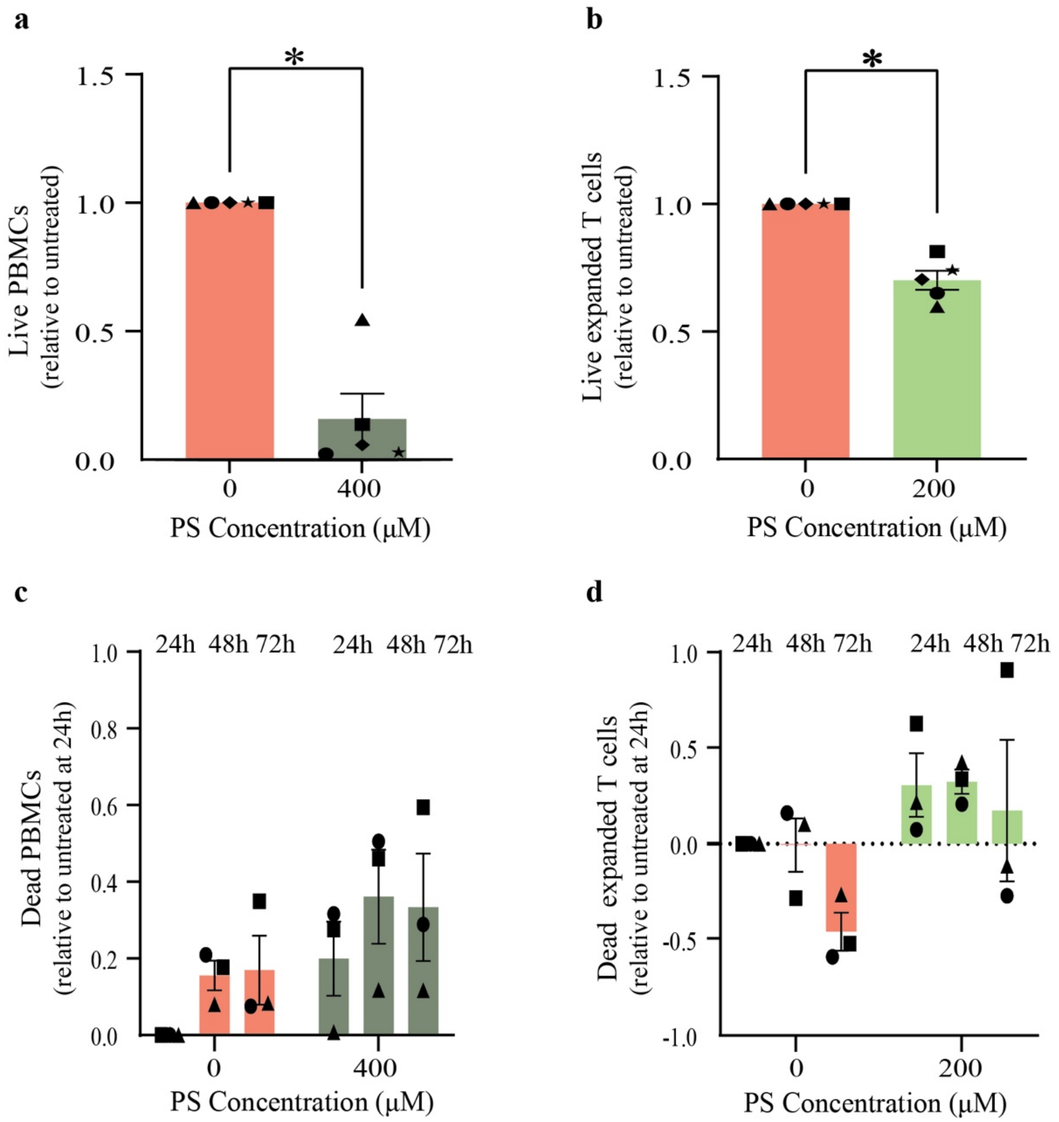

Figure S3. *In vitro* inhibition of T cell viability and apoptosis post-pregnenolone sulfate treatment of non-activated T cells. Normalized bar plots showing the viability of non-activated (**a**) PBMCs and (**b**) expanded T cells across pregnenolone sulfate (PS) treatment concentrations relative to the non-treated, non-activated corresponding samples. Normalized bar plots showing the apoptosis of non-activated (**c**) PBMCs and (**d**) expanded T cells across pregnenolone sulfate treatment concentrations relative to non-activated corresponding samples. Statistical analyses were performed using paired *t*-test for viability assays, and two-way ANOVA for apoptosis test. Experimental design of assays based on outline in Fig. 1a.

Figure S4

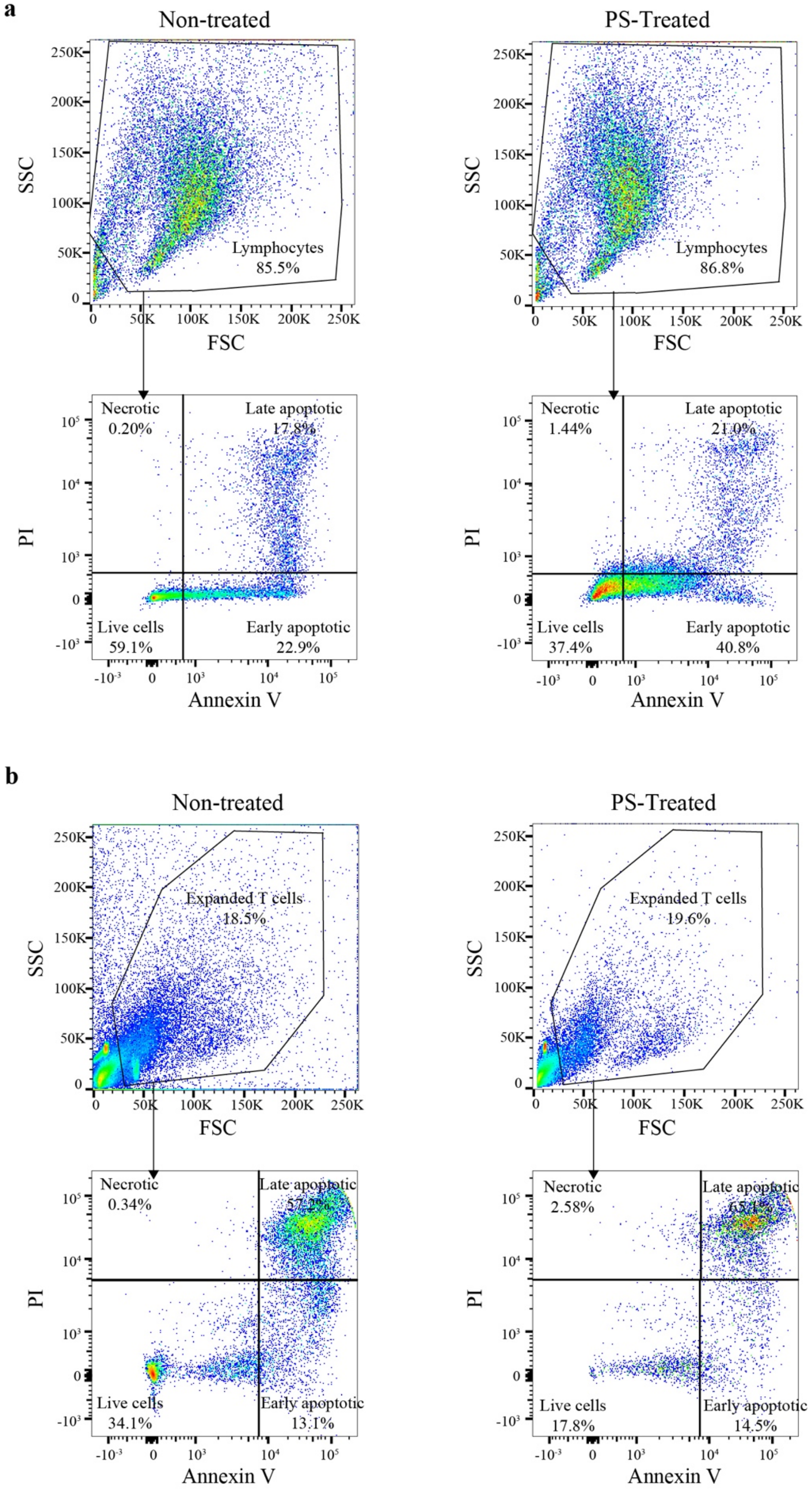
a
Non-treated
PS-Treated
SSC
FSC
Lymphocytes
85.5%
Lymphocytes
86.8%
Necrotic
0.20%
Late apoptotic
17.8%
Live cells
59.1%
Early apoptotic
22.9%
Necrotic
1.44%
Late apoptotic
21.0%
Live cells
37.4%
Early apoptotic
40.8%
PI
Annexin V
b
Non-treated
PS-Treated
Expanded T cells
18.5%
Expanded T cells
19.6%
Necrotic
0.34%
Late apoptotic
57.2%
Live cells
34.1%
Early apoptotic
13.1%
Necrotic
2.58%
Late apoptotic
65.1%
Live cells
17.8%
Early apoptotic
14.5%

Figure S4. Gating strategy used in apoptosis assay. Flow cytometric plots showing the gating strategy used in the non-treated and pregnenolone sulfate (PS)-treated (**a**) PBMCs and (**b**) expanded T cells apoptosis assays from one individual. PBMCs and expanded T cells were gated according to FSC and SSC. Live ($AnnexinV^{-}PI^{-}$), early apoptotic ($AnnexinV^{+}PI^{-}$), late apoptotic ($AnnexinV^{+}PI^{+}$) and necrotic ($AnnexinV^{-}PI^{+}$) lymphocytes were gated according to PI and Annexin-V.

Figure S5

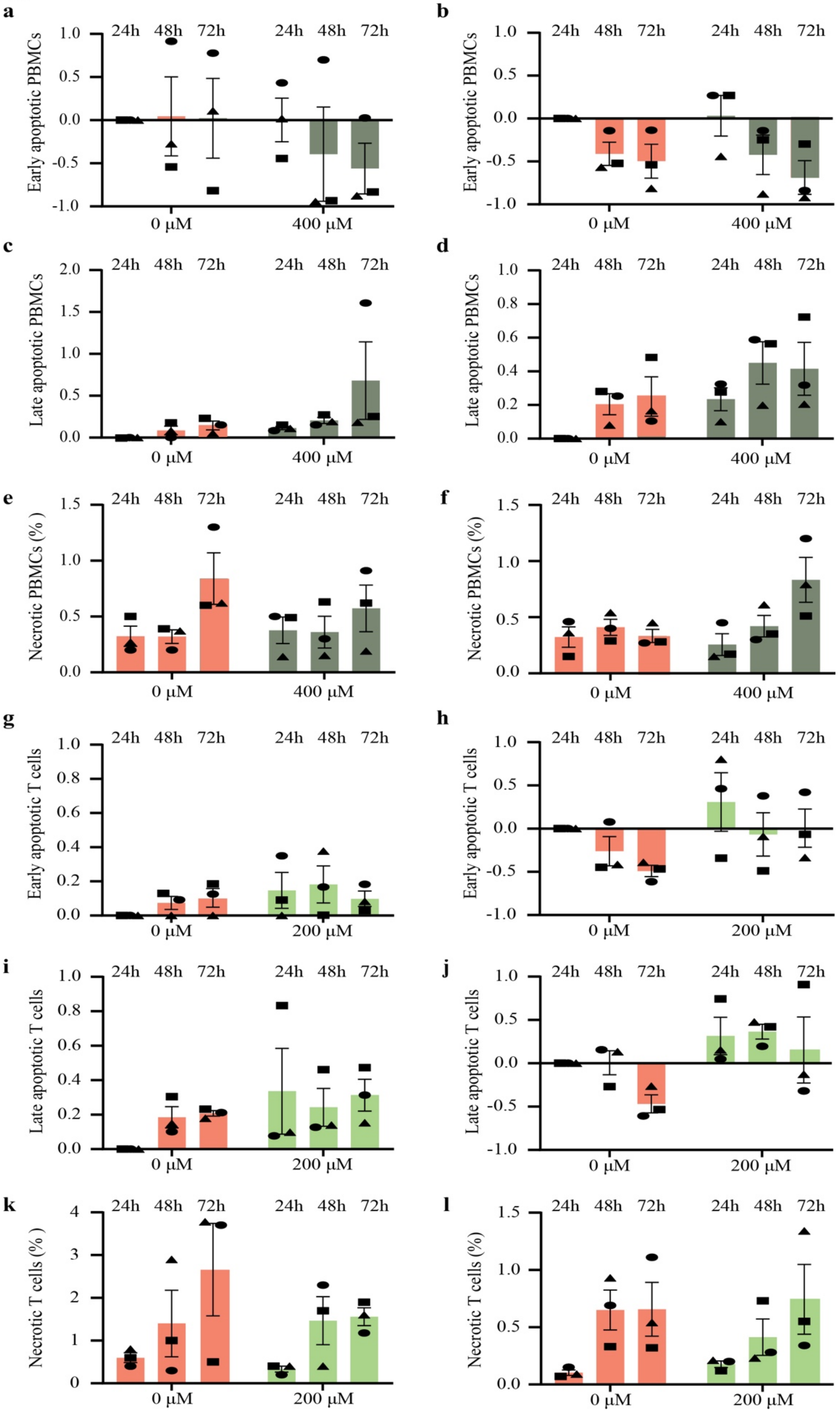
a
b
c
d
e
f
g
h
i
j
k
l
24h 48h 72h 24h 48h 72h
Early apoptotic PBMCs
Late apoptotic PBMCs
Necrotic PBMCs (%)
Early apoptotic T cells
Late apoptotic T cells
Necrotic T cells (%)
0 μM
400 μM
200 μM

Figure S5. ***In vitro*** **induction of apoptotic stages post-pregnenolone sulfate treatment of activated and non-activated PBMCs and expanded T cells**. Normalized bar plots showing the early apoptosis of (**a**) activated and (**b**) non-activated PBMCs, and late apoptosis of (**c**) activated and (**d**) non-activated PBMCs, treated with 200 μM of pregnenolone sulfate relative to non-treated corresponding samples at the 24-hour time point. Percentage of necrotic (**e**) activated and (**f**) non-activated PBMCs are shown here due to their relatively lower number. Normalized bar plots showing the early apoptosis of (**g**) activated and (**h**) non-activated expanded T cells, and late apoptosis of (**i**) activated and (**j**) non-activated expanded T cells treated with 200 μM of pregnenolone sulfate relative to non-treated corresponding samples at the 24-hour time point. The percentage of necrotic (**k**) activated and (**l**) non-activated expanded T cells are shown here due to their relatively lower number. Experimental design of assays based on outline in Fig. 1a.

Figure S6

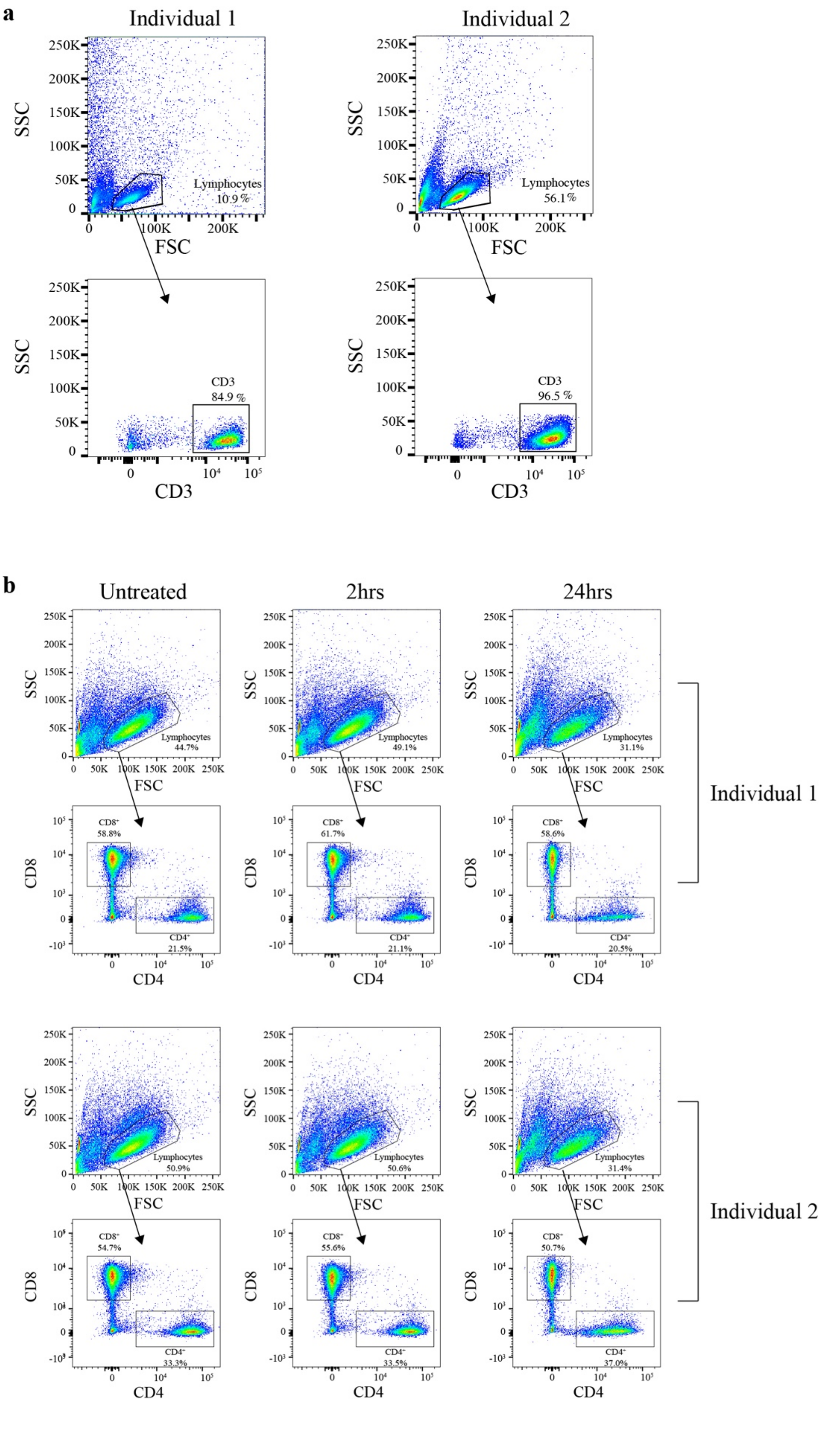
a
Individual 1
Individual 2
Lymphocytes
10.9 %
Lymphocytes
56.1%
SSC
FSC
CD3
84.9 %
CD3
96.5 %
b
Untreated
2hrs
24hrs
Lymphocytes
44.7%
Lymphocytes
49.1%
Lymphocytes
31.1%
CD8⁺
58.8%
CD8⁺
61.7%
CD8⁺
58.6%
CD4⁺
21.5%
CD4⁺
21.1%
CD4⁺
20.5%
Individual 1
Lymphocytes
50.9%
Lymphocytes
50.6%
Lymphocytes
31.4%
CD8⁺
54.7%
CD8⁺
55.6%
CD8⁺
50.7%
CD4⁺
33.3%
CD4⁺
33.5%
CD4⁺
37.0%
Individual 2
CD8
CD4

Figure S6. Immunophenotyping of T cells after expansion for RNA-sequencing experiments. (**a**) Flow cytometry plots showing T cells expanded from PBMCs of 2 donors stained with CD3-PE antibody. (**b**) Flow cytometry plots of expanded T cells from 2 donors that were non-treated, treated for 2 hours or 24 hours with 200 μM of pregnenolone sulfate and stained with CD4-PE and CD8-PB antibodies. Live T cells were primarily gated using FSC and SSC, followed by gating on (**a**) $CD3^+$ cells or (**b**) $CD4^+/CD8^+$ cells.

Figure S7

a

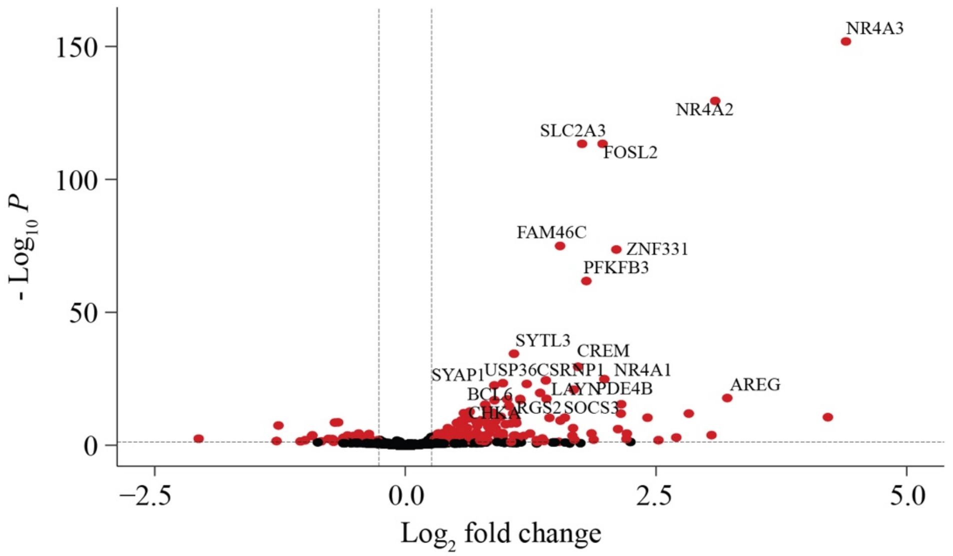

b

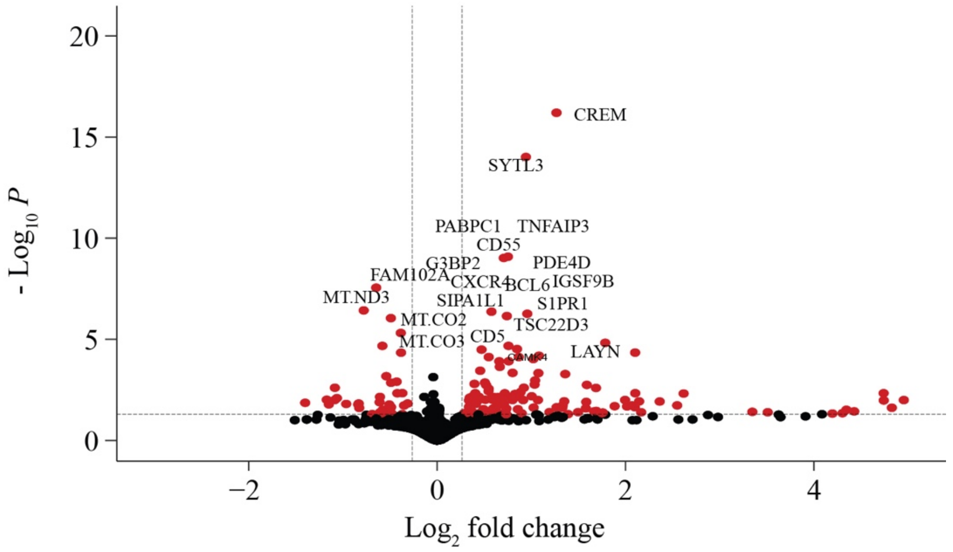

Figure S7. Differential gene expression of pregnenolone sulfate treated and non-treated samples in non-activated T cells. Volcano plots showing differential gene expression before and after pregnenolone sulfate-treatment at (**a**) 2 hours and (**b**) 24 hours post-treatment of the non-activated samples. Significant DEGs after treatment (|FC| ≥ 1.2, B-H FDR < 0.05) are shown in red and the top 20 DEGs are labeled.

Figure S8

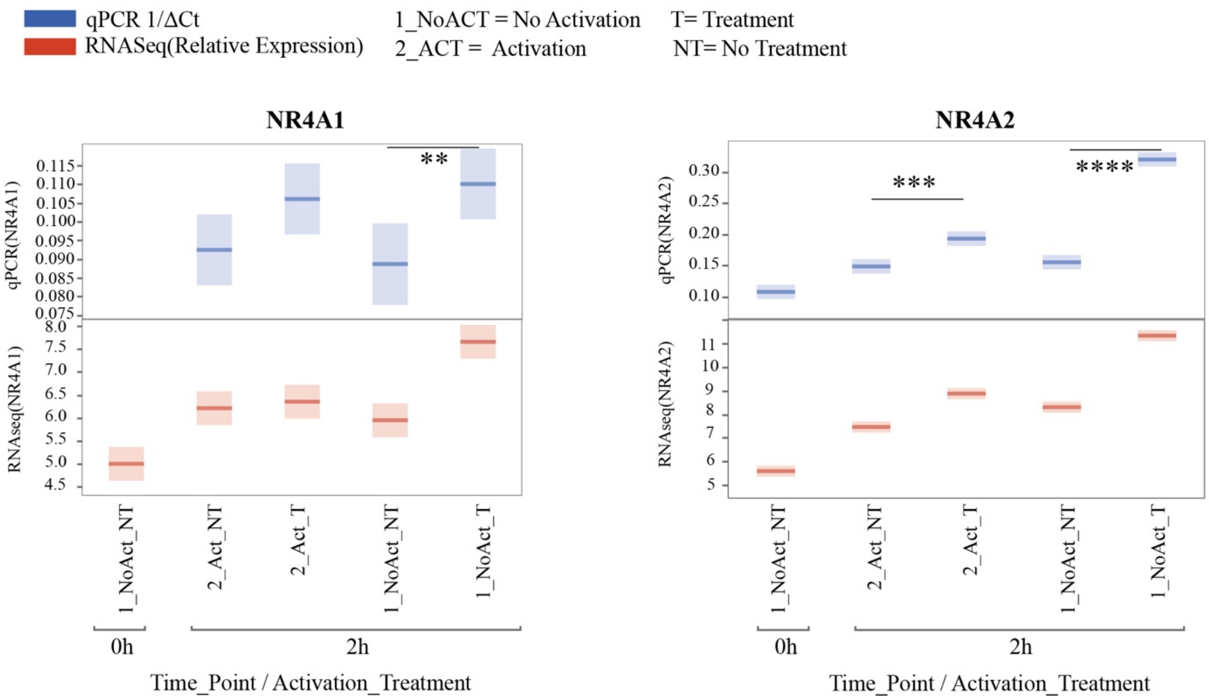

Figure S8. qPCR validation of *NRA41* and *NR4A2* expression. Box plots representing qPCR (1/ΔCt) and RNA-sequencing (VST-normalized expression) expression levels of *NR4A1* and *NR4A2* genes from total RNA of 20 samples (NoACT_NT_0hrs, n = 4; NoACT_NT_2hrs, n = 4; NoACT_T_2hrs, n = 4; ACT_NT_2hrs, n = 4; ACT_T_2hrs, n = 4). Unpaired *t*-test was performed and significance is indicated (* $p < 0.05$, ** $p < 0.01$, *** $p < 0.001$, **** $p < 0.0001$).

Figure S9

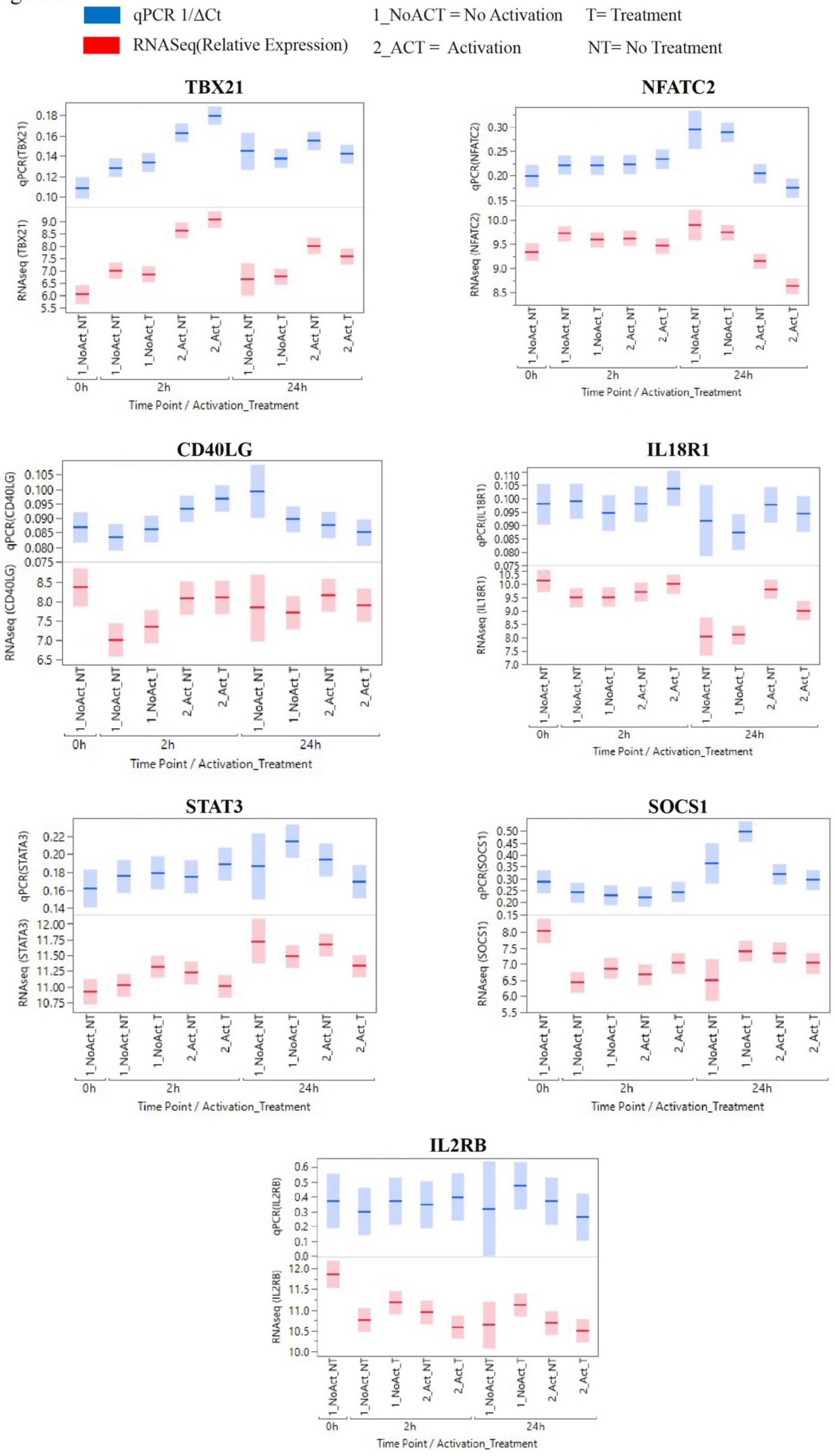
qPCR 1/ΔCt
RNASeq(Relative Expression)
1_NoACT = No Activation
2_ACT = Activation
T= Treatment
NT= No Treatment
TBX21
NFATC2
CD40LG
IL18R1
STAT3
SOCS1
IL2RB
1_NoAct_NT
1_NoAct_T
2_Act_NT
2_Act_T
0h
2h
24h
Time Point / Activation_Treatment

Figure S9. qPCR validation of select differentially expressed genes from RNA-seq. Box plots representing qPCR (1/ΔCt) and RNA-sequencing (VST-normalized expression) expression levels of *TBX1*, *NFATC1*, *CD40LG*, *IL18R1*, *STAT3*, *SOCS1* and *IL2RB* genes from total RNA of 32 samples (NoACT_NT_0hrs, n = 3; NoACT_NT_2hrs, n = 4; NoACT_NT_24hrs, n = 1; NoACT_T_2hrs, n = 4; NoACT_T_24hrs, n = 4; ACT_NT_2hrs, n = 4; ACT_NT_24hrs, n = 4; ACT_T_2hrs, n = 4; ACT_T_24hrs, n = 4).

Figure S10

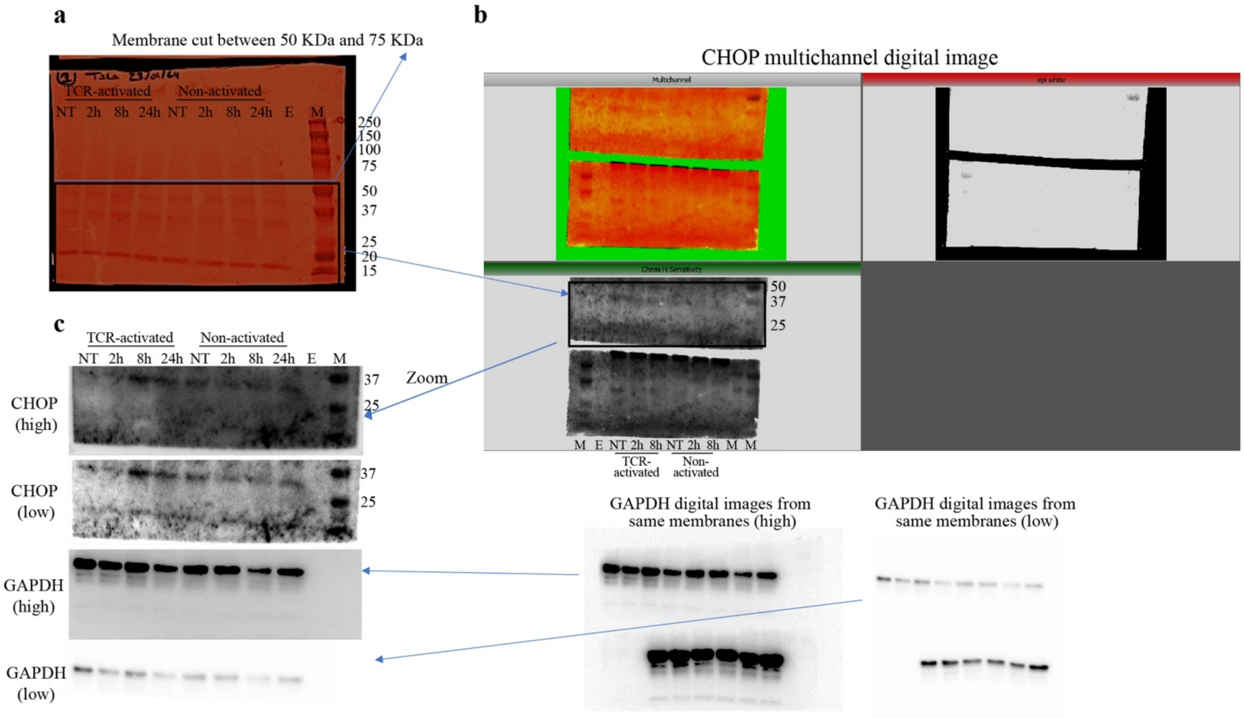

Figure S10. Original Western blots. (**a**) Ponceau stain image of the uncut nitrocellulose membrane used for blotting for CHOP and GAPDH shown in Figure 3b. The box shows where the membrane was cut (between 50 KDa and 75 KDa). The ladder and corresponding sizes in KDa are shown. Whole-cell lysates from expanded T cells of one donor that were TCR-activated (left) or non-activated (right), and either non-treated (NT), or treated with 200µM of pregnenolone sulfate (PS) for 2, 8, or 24 hours, run on a gel and blotted with CHOP rabbit monoclonal antibody, followed by GAPDH mouse monoclonal antibody. M: marker; NT: non-treated; E: empty. (**b**) (Top) Multichannel digital images captured after blotting for CHOP, with the top cut membrane being from (a) and used for Figure 3b, and the bottom cut membrane being a technical replicate (without the 24-hour timepoint). Membrane borders are clearly shown. The blots at the bottom of the panel show high and low exposures after blotting both membranes with GAPDH. (**c**) Images of the first top membrane with high and low exposures of CHOP and GAPDH are shown, which were cropped to show CHOP expression in activated T cells in Figure 3b.

Figure S11

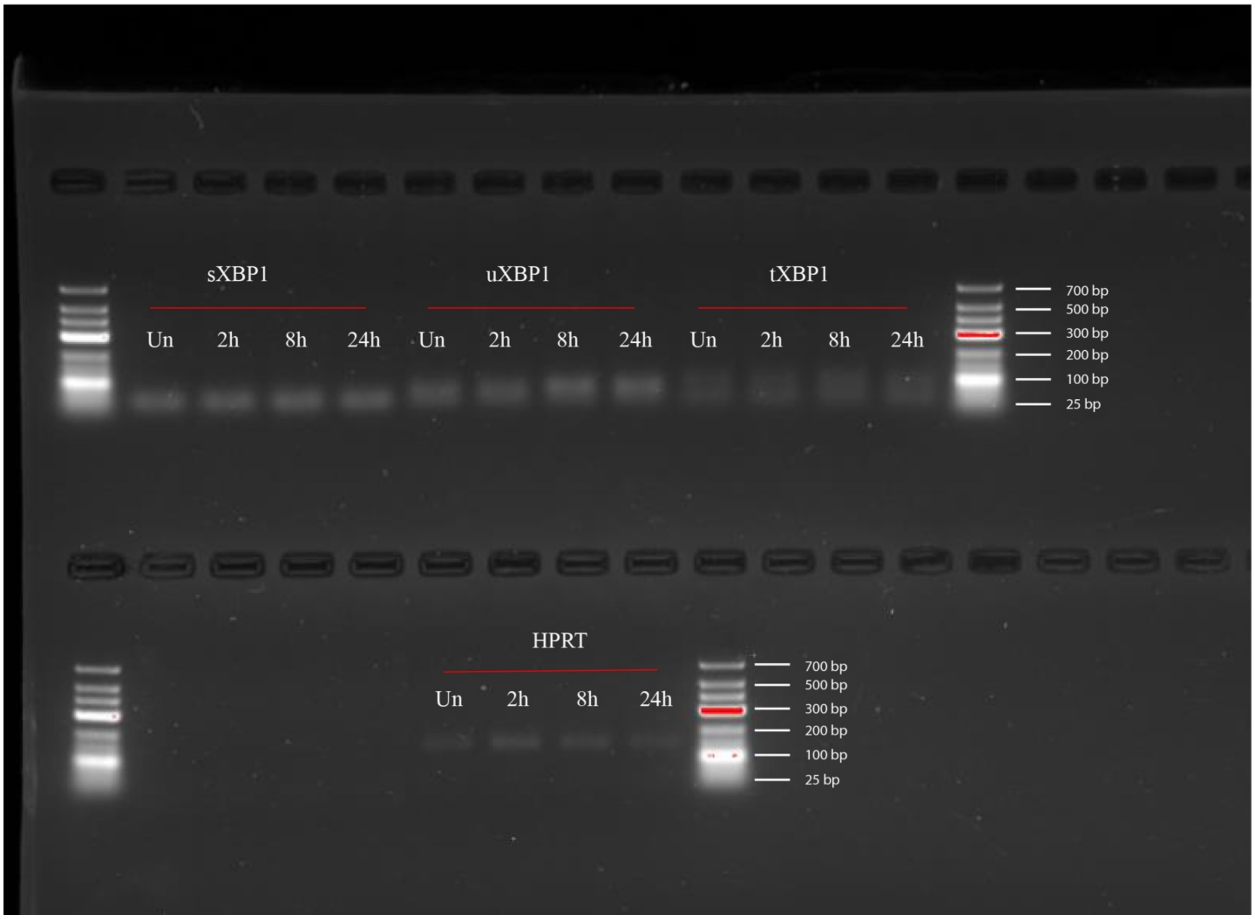

Figure S11. PCR gel for XBP1 splicing. Original uncropped gel image showing bands from PCR amplified cDNA of spliced XBP1 (sXBP1, 59 bp), unspliced XBP1(uXBP1, 76 bp), and total XBP1 (tXBP1, 71 and 97 bp) in the top gel, and HPRT (139 bp) in the bottom gel. PCR amplification was performed on cDNA synthesized mRNA extracted from expanded T cells of one donor that were TCR-activated for 24 hours, followed by no pregnenolone sulfate treatment (Un), or treatment for 2, 8 or 24 hours as indicated.

Figure S12

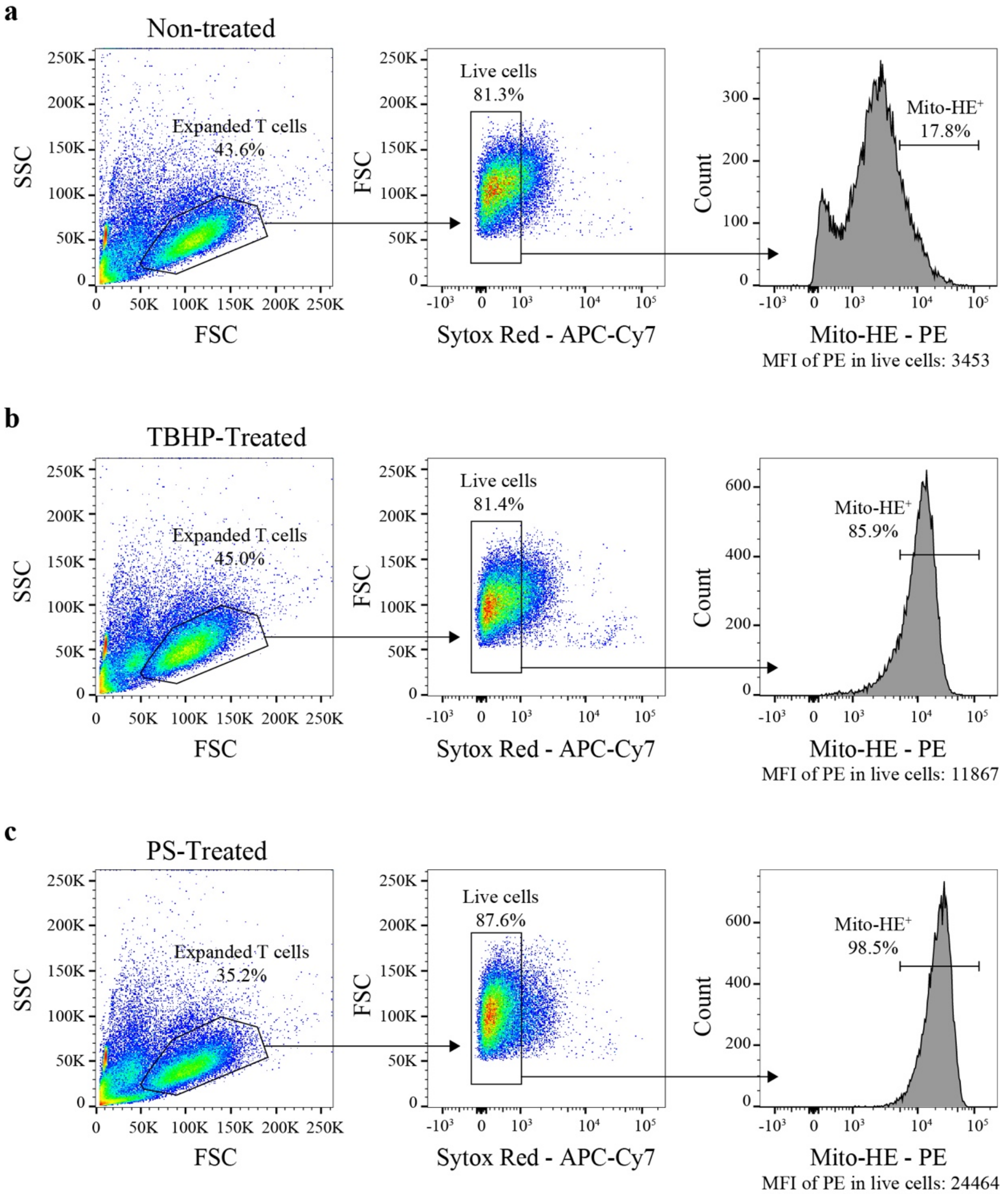

Figure S12. Gating strategy for Mitochondrial superoxide flow cytometric assay. Flow cytometric plots showing the gating strategy used in the (**a**) non-treated, (**b**) tert-Butyl hydroperoxide (TBHP, 250µM)-treated and (**c**) pregnenolone sulfate (PS, 200µM)-treated expanded T cells following mitochondrial superoxide assay from one individual. Expanded T cells were gated according to FSC and SSC, followed by live cell gating using the SYTOX™ Red. Live cells were then gated for Mito-HE and the mean fluorescence intensity (MFI) of the Mito-HE dye within the live cell gate was recorded by FlowJo™.
